# Sensitivity analysis of natural convection in a porous cavity filled with nanofluid and equipped with horizontal fins using various optimization methods and MRT-LB

**DOI:** 10.1038/s41598-024-60330-0

**Published:** 2024-04-29

**Authors:** H. Sajjadi, N. Mansouri, S. N. Nabavi, A. Amiri Delouei, M. Atashafrooz

**Affiliations:** 1 Department of Mechanical Engineering, University of Bojnord, Bojnord, Iran; 2 Department of Mechanical Engineering, Iran University of Science and Technology, Tehran, Iran; 3 Department of Mechanical Engineering, Sirjan University of Technology, Sirjan, Iran

**Keywords:** Natural convection, Porous media, Nanofluid, Multi-relaxation time lattice Boltzmann method, Taguchi method, Response surface method, Genetic algorithm, Energy science and technology, Engineering, Mathematics and computing, Nanoscience and technology

## Abstract

In the present study, natural convection heat transfer is investigated in a porous cavity filled with Cu/water nanofluid and equipped with horizontal fins. Optimization and sensitivity analysis of the fin’s geometry, porous medium and nanofluid properties to maximize heat transfer rate is the aim of this work. To achieve this purpose, a design space is created by input parameters which include length, number of fins, distance between fins, porosity, Darcy number and volumetric fraction of the nanoparticles. Several tools have been used to implement optimization methods including the Taguchi method (TM) for design points generation, sensitivity analysis of design variables by using signal-to-noise ratio (SNR) and analysis of variance (ANOVA), response surface method (RSM) for interpolation and regression by using nonparametric regression, and genetic algorithm (GA) for finding optimum design point. The double multi-relaxation time lattice Boltzmann method (MRT-LBM) is used to analyze and simulate the flow field and heat transfer in each design point. The results show that the optimal configuration leads to an average Nusselt number of 5.56. This optimal configuration is at the length of fins L/2, the number of fins 2, the distance between fins L/12, porosity 0.8, Darcy number 0.1, and the volumetric fraction of the nanoparticles 0.02. By using the SNR results, the Darcy number and the number of fins have the most and the least effect in maximizing the average Nusselt number, respectively. The ANOVA results and global sensitivity analysis (GSA) findings further validated this conclusion.

## Introduction

Natural convection heat transfer has many applications in different areas like electronic cooling, solar systems, heating, ventilation and air conditioning, etc. Since the natural convection heat transfer coefficient is very low, various techniques are offered to increase this defect. One appropriate way to increase the heat transfer coefficient is to use porous material. These structures by having large heat transfer areas improve the heat transfer rate between fluid and solid. Although porous media has superb thermal efficiency, they have low thermal conductivity. To compensate for this deficiency, two solutions can be applied, using extended surfaces like fins and baffles or adding nanoparticles with a higher thermal conductivity.

There have been done various numerical simulation to investigate the effect of porous media, nanofluids and fins on natural convection heat transfer^1–11^. In many of these studies square cavity was utilized to peruse fluid flow, heat transfer, mass transfer and interaction between them. Hossein et al.^12^ investigated the effect of the length and distance between fins on heat transfer and fluid flow in a cavity with CU/water nanofluids and showed that the existence of fins has a great effect on heat transfer and fluid flow in the cavity. The effect of the Hartmann number on fluid flow and heat transfer in the cavity without and with fins has also been studied. Al-Farhany et al.^13^ investigated the impact of length and distance between fins on conjugate natural convection in a porous cavity. Their results showed that the Nusselt number increases with the increase of Rayleigh number, Darcy number and fin length. The results also indicated that the greater distance between the fins when the fins are at their maximum length is the best structure for heat transfer. Arani and Roohi^14^ investigated natural convection heat transfer in a square cavity with a baffle filled with two types of copper and aluminum nanofluids. Their results showed that due to the higher heat transfer coefficient of copper conductivity, the effect of adding these particles on the heat transfer rate in this case is much higher than the presence of aluminum oxide particles. Mahmoodi and Asef^15^ also studied natural convection heat transfer in a square cavity with a baffle filled with Cu/water nanofluid. They investigated the effect of baffle position, Rayleigh number and volume fraction of nanoparticles. Their results showed that the appropriate position of the baffle is different in various Rayleigh numbers, and where Ra = 1000, the heat transfer rate is maximized when the baffle is in the center of the hot wall. Hamida and Hatami^16^ compared the position of fins in a channel filled with mixed nanofluid and under the effect of an electric field. The number of fins was 4–8, and the distance between them was 2–4 cm and they used Galkin finite element method to investigate the problem. The results showed that the maximum Nusselt number happened when hybrid titanium oxide-aluminum oxide with 0.05 volume fraction was used.

Ahmed et al.^17^ investigated the effect of some parameters including nanoparticle volume fraction, Darcy number, number of undulation and amplitude of waviness for a wavy walled porous cavity filled with Cu-Al_2_O_3_/water hybrid nanofluid. The results showed that the Darcy number is an important parameter on fluid flow and temperature. They also showed that the heat transfer rate increased by increasing nanoparticle volume fraction. Their result illustrated that the growth in the undulation and wavy contraction ratio causes a decrease in the fluid flow while it increases the local Nusselt number on the wavy wall. Izadi et al.^18^ used a periodic magnetic field on a hybrid nanofluid in the porous cavity. They concluded that magnetic field inclination angle and periodical magnetic field wave length can control heat transfer performance within the liquid and solid phases. The effect of magnetohydrodynamics forced convection of nanofluid on heat transfer in a U-shaped cavity with a porous region and wavy wall was studied by Selimefendigil and Öztop^19^. The rise of magnetic field strength increased the average Nusselt number significantly. However, the impact is reduced by a higher Darcy number of the porous domain. Baghsaz et al.^20^ studied the effect of nanoparticle sedimentation on characteristics of natural convection over time. They reported that the Nusselt number decreased during the nanoparticle sedimentation process.

Also, due to the widespread capability of the Lattice Boltzmann method (LBM) in the analysis of fluid flow and heat transfer, this methodology has garnered significant attention from numerous scholars to investigate the nanofluids flow and flow in porous mediums^21–24^. Sajjadi et al.^25^ studied natural convection heat transfer in the porous cavity by using the double multi relaxation time Lattice Boltzmann method (MRT-LBM). They showed that the heat transfer rate increases with the rise of Darcy number, porosity, Rayleigh number and volume fraction of nanoparticles. It was also observed that the effect of Darcy number on the heat transfer rate increases with the increase of Rayleigh number. Sheikholeslami and Vajaraulu^26^ used the LBM to investigate the effect of a magnetic field on free convection heat transfer in a porous cavity filled with nanofluids. The results showed that the temperature gradient decreases with the increase of the Hartmann number. MRT-LBM was utilized by Rahimi et al.^27^ to investigate the effects of Rayleigh number, solid volume fraction, and four different arrangements of discrete active walls on the natural convection and entropy generation in the three-dimensional domain. They reported that the location of active walls has considerable effects on heat transfer performance, also Rayleigh number and solid volume fraction enhanced the average Nusselt number. Their results illustrate that increasing the Rayleigh number leads to higher entropy generation, while the increasing the solid volume fraction decreases entropy generation.

It can be beneficial to use the design of experiments (DOE) to reduce the costs of studying parameters and statistical and metamodeling methods to check the effect of different parameters and optimization. Taguchi method (TM) and response surface method (RSM) are two useful models in this regard. The TM, which was introduced by Taguchi^28^, combines statistical and mathematical techniques to design experiments, investigate the influence of variables on response and find optimum points. Sobhani et al.^22^ investigated the radiation/natural convection in a cavity with a horizontal fin. Their results showed that the length and location of the fin have fewer effect compared to some operational variables like Rayleigh number. Hydrodynamics and heat transfer in a microchannel heat sink are studied by Bazkhane and Zahmatkesh^29^. Javadpour^30^ optimized microchannel performance filled by nanofluid using TM and genetic algorithm (GA). RSM, which was introduced by Box and Wilson^31^ is a metamodeling technique that is developed as a surrogate of the expensive simulation process to improve the overall computation efficiency. To minimize substantial computational cost, RSM enables the manipulation of a continuous response space based on the given design parameters and provides an opportunity to evaluate numerous design cases and assess the impact of the designated factors. This method widely applied for optimization in different types of issues as a useful outcome. For example, RSM was used for the optimization of natural ventilation in a building^32^, the configuration of a spacer in a spiral-wound membrane^33^, the membrane-based separation process of isopropanol/water solution^34^, etc.

To the best of the authors’ knowledge, no previous work has used combined LBM and RSM methods to assess and optimize the heat transfer of nanofluid in porous cavities with fins. The primary objective of this study is to conduct a sensitivity analysis and optimize the process of natural convection heat transfer. Therefore, the design points were selected using the TM, and MRT-LBM was applied to simulate the physics of fluid. Six variables including length, number and distance between fins, porosity, Darcy number and volumetric fraction of nanoparticles are considered as input factors and accordingly, the output parameter of the Nusselt number has been evaluated. Signal to noise ratio (SNR), analysis of variance (ANOVA), and the global sensitivity analysis (GSA) have been employed to assess the sensitivity of the system. In the concluding phase of this study, the optimal design point will be identified through the utilization of the TM and GA. Subsequently, a comparative analysis of the results will be conducted.

## Methodology

### Present geometry and governing equations

**Figure 1:**
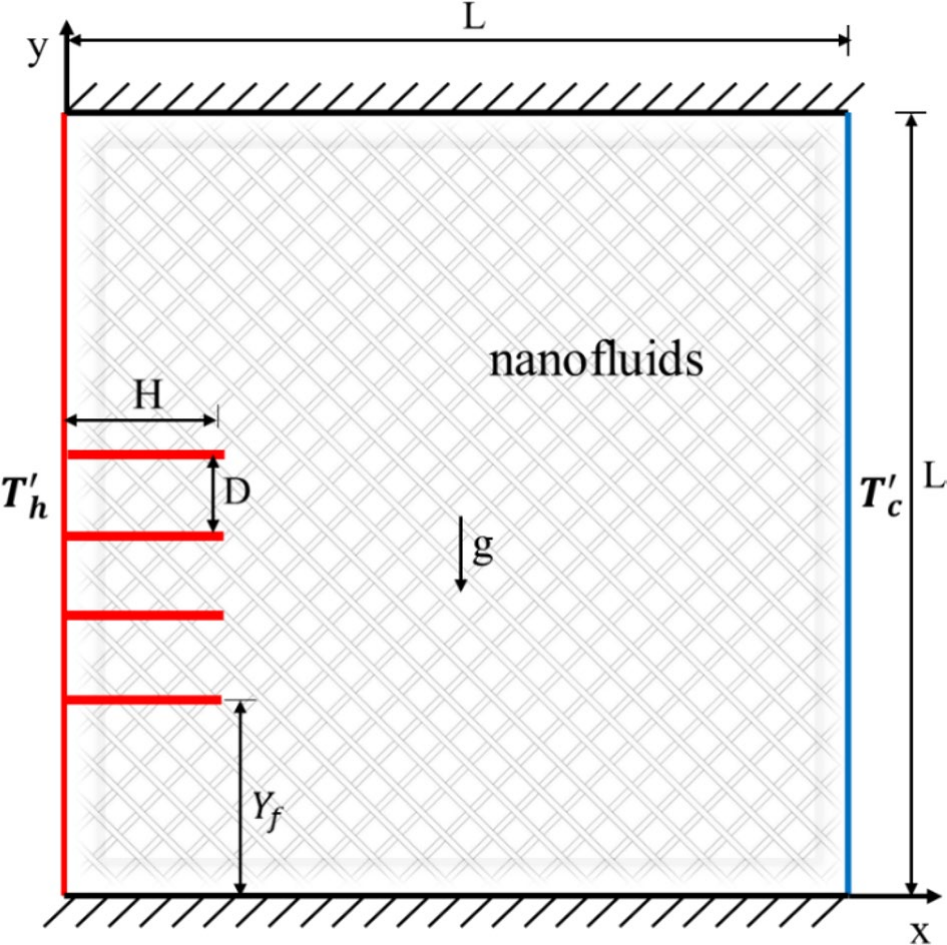
Geometry of the problem.

In this research, a two-dimensional porous enclosure was utilized to investigate the behavior of fluid flow and heat transfer (Fig. 1). As shown in Fig. 1, the left side of the cavity and fins have a constant temperature of ${T}_{h}$, the right side has a constant temperature of ${T}_{c}$ and the top and bottom walls are adiabatic. All walls and fins are fixed and non-slip boundary condition is used for them. The physical and thermal boundary conditions can be summarized as follows:

- At the hot wall of the cavity and the fins surface: $U=V=0, {T}^{\prime}= {T}_{h}^{\prime}$
- At the cold wall of the cavity: $U=V=0, {T}^{\prime}= {T}_{c}^{\prime}$
- At the insulation walls: $U=V=0, \partial T/\partial y=0$

Also, L is equal to unity. ${Y}_{f}$ is the distance between the first fin and south wall which is fixed and equal to L/4. $H$ is the length of fins and $D$ is the distance between fins.The enclosure contains Cu/water, which is treated as a Newtonian fluid. The flow is characterized as laminar, and the effects of viscous heat dissipation are not taken into account. So, the fundamental equations for continuity, momentum, and energy are provided in the following dimensionless equations, respectively^19^:

1 $$\frac{\partial U}{\partial X}+\frac{\partial V}{\partial Y}=0,$$

2 $$\frac{\partial U}{\partial t}+U\frac{\partial U}{\partial X}+V\frac{\partial U}{\partial Y}=-\frac{\partial P}{\partial X}+{\text{Pr}}\left(\frac{{\partial }^{2}U}{\partial {X}^{2}}+\frac{{\partial }^{2}U}{\partial {Y}^{2}}\right)-\frac{\varepsilon {\text{Pr}}}{{\text{Da}}}U-\frac{1.75}{\sqrt{150\varepsilon {\text{Da}}}}U\sqrt{{\left|U\right|}^{2}+{\left|V\right|}^{2}}$$

3 $$\frac{\partial V}{\partial t}+U\frac{\partial V}{\partial X}+V\frac{\partial V}{\partial Y}=-\frac{\partial P}{\partial Y}+{\text{Pr}}\left(\frac{{\partial }^{2}V}{\partial {X}^{2}}+\frac{{\partial }^{2}V}{\partial {Y}^{2}}\right)-\frac{\varepsilon {\text{Pr}}}{{\text{Da}}}V-\frac{1.75}{\sqrt{150\varepsilon {\text{Da}}}}V\sqrt{{\left|U\right|}^{2}+{\left|V\right|}^{2}}+\varepsilon {\text{RaPr}}T$$

4 $$\frac{\partial T}{\partial t}+\varepsilon U\frac{\partial T}{\partial X}+\varepsilon V\frac{\partial T}{\partial Y}=\left(\frac{{\partial }^{2}T}{\partial {X}^{2}}+\frac{{\partial }^{2}T}{\partial {Y}^{2}}\right)$$

In Eq. 2–4$(\varepsilon )$ is porosity which will be calculated in below equation:

5 $$\varepsilon =\frac{{V}_{void}}{{V}_{total}}$$

Which ${V}_{void}$ is volume of voids and ${V}_{total}$ is total volume.

To obtain above dimensionless equations, the following variables were used:

6 $$X=\frac{x}{L} ;\quad Y=\frac{y}{L};\quad U=\frac{uL}{\varepsilon \alpha } ;\quad  V=\frac{vL}{\varepsilon \alpha } ;\quad  T=\frac{{T}^{\prime}-{T}_{c}^{\prime}}{{T}_{h}^{\prime}-{T}_{c}^{\prime}} ;\quad P=\frac{p{L}^{2}}{{\rho \alpha }^{2} };\quad {\text{Da}}=\frac{K}{{L}^{2}};\quad {\text{Pr}}=\frac{\vartheta }{\alpha };\quad {\text{Ra}}=\frac{g\beta \left({T}^{\prime}-{T}_{ref}\right){L}^{3}}{\vartheta \alpha }$$

### MRT-LBM for flow field

**Figure 2:**
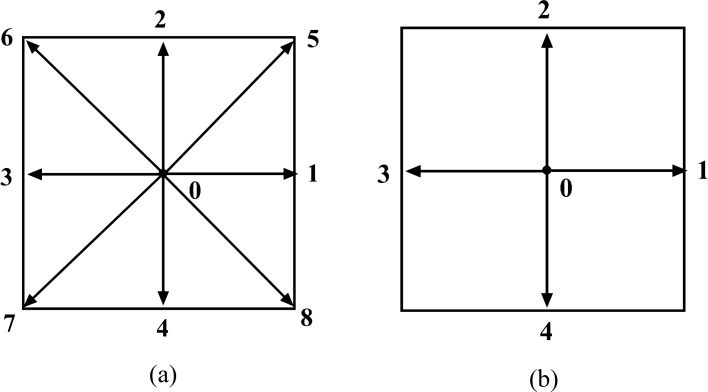
The discrete velocity vectors for (**a**) D2Q9 (**b**) D2Q5.

For solving the collision step in LBM two main model have been presented; 1. Bhatnagar-Gross-Krook (BGK) model, which is the simplest collision model, where the distribution functions are relaxed towards a local equilibrium distribution at each lattice point. The relaxation time parameter controls the rate at which the distribution functions approach equilibrium. 2. Multiple Relaxation Time (MRT) model: in this model, the collision step is decomposed into multiple relaxation processes, allowing for more flexibility and control over the relaxation rates of different moments of the distribution functions which is more stable. So, in this study the MRT model with D2Q9 lattice (Fig. 2a) is used to solve the velocity field. For incompressible fluid in this method, we have^19^:

7 $${f}_{i}\left(x+{c}_{i}\Delta t, t+\Delta t\right)={f}_{i}\left(x,t\right)-{{M}_{ij}}^{-1}.{S}_{jk}.\left[{m}_{k}\left(x,t\right)-{m}_{k}^{eq}\left(x,t\right)\right]+{F}_{i}$$

In Eq. (7) ${f}_{i}$ is the velocity distribution function, $\Delta t$ is the time step which is considered equal to the unity, ${c}_{i}$ is the discrete fluid particle velocity vector for D2Q9 model that is defined in Fig. 2a and reported as:

8 $${c}_{i}=\left\{\begin{array}{ll}\begin{array}{l}0,\\ c \left(\pm \mathrm{1,0}\right),\\ c \left(0,\pm 1\right),\\ c \left(\pm 1,\pm 1\right),\end{array}& \begin{array}{l}\begin{array}{l}i=0,\\ i=\mathrm{1,3},\end{array}\\ \begin{array}{l} i=\mathrm{2,4},\\ i=\mathrm{5,6},\mathrm{7,8},\end{array}\end{array}\end{array}\right.$$

where c is the lattice speed and equals to ∆x/∆t which also set equal to unity.

9 $$M=\left[\begin{array}{ccccccccc}1& 1& 1& 1& 1& 1& 1& 1& 1\\ -4& -1& -1& -1& -1& 2& 2& 1& 2\\ 4& -2& -2& -2& -2& 1& 1& 1& 1\\ 0& 1& 0& -1& 0& 1& 0& -1& 1\\ 0& -2& 0& 2& 0& 1& -1& -1& 1\\ 0& 0& 1& 0& -1& 1& 1& -1& -1\\ 0& 0& -2& 0& 2& 1& 1& -1& -1\\ 0& 1& -1& -1& 1& 0& 0& 0& 0\\ 0& 0& 0& 0& 0& 1& -1& 1& -1\end{array}\right]$$

${M}_{ij}$ is the transformation matrix, ${S}_{jk}$ is the diagonal matrix for relaxation rate for momentum distribution which is calculate as follow:

10 $${S}_{jk}=diag({s}_{0},{s}_{1},\dots {,s}_{8})$$

11 $${s}_{\mathrm{0,3},5}=1, {s}_{\mathrm{7,8}}=\frac{1}{{\tau }_{v}}, {s}_{\mathrm{1,2}}=1.1, {s}_{\mathrm{4,6}}=1.2,$$

Which ${\tau }_{v}$ in Eqs. (11) can be obtained as:

12 $${\tau }_{v}=3v+0.5$$

### MRT Lattice Boltzmann method for temperature field

The MRT Lattice Boltzmann method is employed in this study to solve the distribution function of temperature, similar to the flow field. Additionally, a D2Q5 lattice, as depicted in Fig. 2b, is utilized^16^.

13 $${h}_{i}\left(x+{e}_{i}\Delta t, t+\Delta t\right)={h}_{i}\left(x,t\right)-{{N}_{ij}}^{-1}.{Q}_{jk}.\left[{n}_{k}\left(x,t\right)-{n}_{k}^{eq}\left(x,t\right)\right]$$

Here ${h}_{i}$ is the distribution function for the temperature and ${e}_{i}$ is the discrete fluid particle velocity vector for D2Q5 (Fig. 2b) and is defined as:

14 $${e}_{i}=\left\{\begin{array}{ll}0,& i=0,\\ c\left(\pm \mathrm{1,0}\right),& i=\mathrm{1,3},\\ c\left(0,\pm \right),& i=\mathrm{2,5},\end{array}\right.$$

And values of ${N}_{ij}$ is the transformation matrix and ${Q}_{jk}$ is the diagonal matrix which are can be obtained by:

15 $$N=\left[\begin{array}{ccccc}1& 1& 1& 1& 1\\ 0& 1& 0& -1& 0\\ 0& 0& 1& 0& -1\\ 4& 1& 1& 1& 1\\ 0& 1& -1& 1& -1\end{array}\right]$$

16 $${Q}_{ij}={\text{diag}}\left({q}_{0}, {q}_{1},\cdots ,{q}_{4}\right)$$

Components of the matrix of relaxation time for the present study are defined as:

17 $${q}_{0}=1, {q}_{\mathrm{1,2}}=\frac{1}{{\tau }_{T}}, {q}_{\mathrm{3,4}}=1.5,$$

### Lattice Boltzmann method for nanofluid

Due to the presence of nanoparticles in the base fluid, the thermophysical properties of the nanofluid are different from the base fluid (Table 1). Thermal expansion coefficient (β) of nanofluid is obtained from the following equation:

**Table 1:** Thermophysical properties of water and copper nanoparticles ^25^.

Property	Unit	Water	Copper
Density $(\rho )$	Kg m^−3^	997	8954
Specific heat capacity $({C}_{p})$	J kg^−1^ K^−1^	4179	383
Thermal conductivity $(k)$	W m^−1^ K^−1^	0.6	400
Thermal expansion coefficient $(\beta )$	K^−1^	2.1 × 10^−4^	1.67 × 10^−5^
Dynamic viscosity $(\mu )$	Kg m^−1^ s^−1^	8 × 10^−4^	–

18 $${(\rho \beta )}_{nf}=\left(1-\phi \right){\left(\rho \beta \right)}_{f}+\phi {(\rho \beta )}_{{s}^{\prime}}$$

The heat capacitance (${c}_{p}$) and the effective density of the nanofluid are calculate:

19 $${(\rho {c}_{p})}_{nf}=\left(1-\phi \right){\left(\rho {c}_{p}\right)}_{f}+\phi {(\rho {c}_{p})}_{{s}^{\prime}}$$

20 $${\rho }_{nf}=\left(1-\varphi \right){\rho }_{f}+\varphi {\rho }_{s}$$

Also, the dynamic viscosity of the nanofluid is obtained as

21 $${\mu }_{nf}=\frac{{\mu }_{f}}{{(1-\phi )}^{2.5}}$$

The effective thermal conductivity coefficient is also calculated from Eq. (22).

22 $$\frac{{k}_{nf}}{{k}_{f}}=\frac{{k}_{s}+2{k}_{f}-2\phi ({k}_{f}-{k}_{s})}{{k}_{s}+2{k}_{f}+2\phi ({k}_{f}-{k}_{s})}$$

where in Eqs. (18)–(22), $\phi$ is the volume fraction of the solid particles and subscripts $s$, $nf$ and $f$ are used for solid, nanofluid and base fluid, respectively.

## Taguchi method (TM) and response surface method (RSM)

Geometric conditions and physical parameters can affect the output of issues. Studying the design variables separately causes the effect of changing different parameters simultaneously on the outputs of the problem to be ignored. Also, using full factorial analysis in design space is hugely time-consuming, especially for numerical methods which need considerable computational time and cost for each simulation. Owing to this fact, in this study, the TM and GA have been applied to investigate complex interactions between parameters and obtain outputs in the whole design space. This framework is conducted in four main steps:

In the first step, the input and output would be defined and the design of experiments (DOE) scheme will be implemented on input parameters to achieve design points effectively. The cost of parameters study and the chance of having overlapping data points decrease significantly by employing DOE. Here, the TM is utilized to design experiments for six input factors and in three levels. Taguchi suggested the L_27_ orthogonal arrays table for this design space which are initial design points.

In step 2, numerical simulations based on MRT-LBM discussed in sections "MRT Lattice Boltzmann method for temperature field"–2.5 will be performed to calculate the response of design points.

In step 3, the obtained results of the previous step will be used to predict a relationship between variables and create a continuous space with a response that will give outputs at each point in the design space. The TM also uses a factor called SNR to express the quality of optimization problem and evaluate the performance of each experiment. The value of SNR expresses the dispersion of the simulated data around the nominal or target value by using the loss function. The SNR is divided into three types: the smaller the better, the larger the better, and the nominal the better, thus the calculation is different. In the present study, because the purpose is to enhance the output parameter (average Nusselt number), the larger the better type will be used which is computed according to as follows,

23 $${SN}_{LB}=-10{\text{log}}\left(\frac{1}{n} \sum_{i=1}^{n}\frac{1}{{{y}_{i}}^{2}}\right)$$

where $n$ is the number of iteration experiments and ${y}_{i}$ is the output of the problem. To determine the effective variables and their contribution on the performance of the objectives an ANOVA is used.

Although valuable results can be obtained from the TM, this method is unable to predict the result in all design spaces. According to this fact, RSM will be applied to predict results in all domains. For this reason, some samples add to initial design points to enrich design space and obtain better results. Then by carrying non-parametric regression (NPR) a continuous design space will be created. NPR is a metamodeling technique that provides improved response quality. NPR belongs to a general class of Support Vector Method (SVM) type techniques. This model revolves around the concept of forming a narrow envelope, known as epsilon, around the output response surface and extending it. This envelope should encompass all or most of the design sample points. This method is one of the approaches that is more flexible, data-driven and suitable for noisy results compared to other metamodeling techniques like standard response surface and kriging model.

Finally, in step 4, optimum conditions will be achieved using GA and the optimum point achieved from TM optimization will be comparied by optimum point of GA.

### Input and output parameters

The number of fins, the length of them and the distance between them are three main geometrical parameters related to fins, porosity and Darcy number are other geometrical parameters related to structure of the porous medium and volumetric fraction of the nanoparticles is an operational variable related to nanofluids. The number of fins is a discrete parameter which in the present study are 2, 3 and 4, the length of fins is L/6, L/3 and L/2, and distance between them is L/6, L/8 and L/12, porosity is 0.4, 0.6 and 0.8, volumetric fraction of the nanoparticles is 0.005, 0.01 and 0.02. The Darcy number is a dimensionless number which is normally used in heat transfer through porous media and equal to below equation:

24 $${\text{Da}}= \frac{k}{{d}^{2}}$$

where $k$ is the permeability, which is capability of passing the fluid through the porous media, and $d$ is the characteristic length. The Dary number is 0.001, 0.01 and 0.1 in the present work.

Average Nusselt number on the cold wall is the only output of this study and calculated as:

25 $${\text{Nu}}=\frac{L}{\Delta \theta }{\left.\frac{-\partial \theta }{\partial x}\right|}_{x=L}$$

26 $${{\text{Nu}}}_{avg}={\int }_{0}^{L}{\text{Nu}}dy$$

## Validation and grid independence

**Figure 3:**
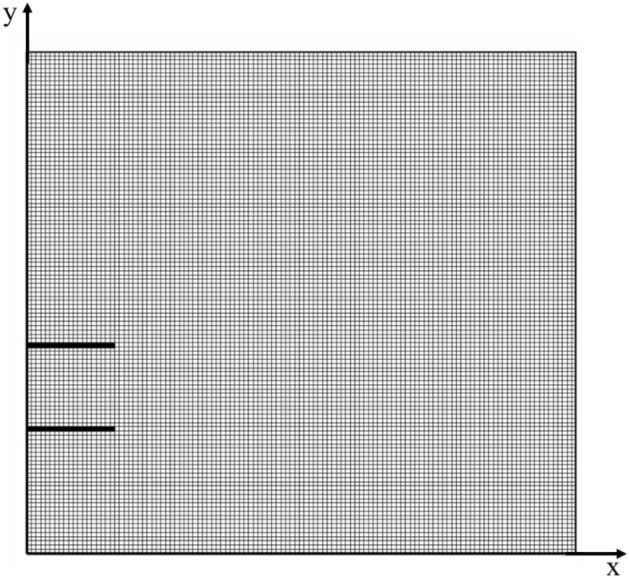
Grid distribution for N3.

A thorough testing process was carried out to ensure that the solution was not dependent on grid size. The average Nusselt number on the cold wall for specific conditions (DP = 1 in Table 5) was calculated at various grid resolutions and displayed in Table 2. As shown in Fig. 3. Uniform grid has been applied in the present study. The results indicated that there was not a significant change in the average Nusselt number as the grid resolution increased from N3 to N4, leading to the selection of a (120 × 120) grid resolution.

**Table 2:** Grid independence study.

	N1	N2	N3	N4
Grid size	80 × 80	100 × 100	120 × 120	130 × 130
Nu	2.015	2.095	2.122	2.121

To ensure the accuracy of the current work, validation was conducted in two areas by comparing it with previous research. Initially, the nanofluid solution was validated against the findings of Khanafer et al.^35^ as depicted in Table 3. The present approach successfully replicates the results for the nanofluid with a maximum error of under 5%, as shown in Table 3.

**Table 3:** The average Nusselt number for different nanoparticle volumetric fractions in comparison to the prior study^35^.

	Ra = 10^3^			Ra = 10^4^			Ra = 10^5^		
	Present work	Khanafer et al.^29^	Error (%)	Present work	Khanafer et al.^29^	Error (%)	Present work	Khanafer et al.^29^	Error (%)
0%	1.87	1.96	0.45	4.05	4.03	0.49	8.13	8.31	2.1
4%	2.07	2.06	0.48	4.39	4.36	0.68	8.8	8.91	1.2
8%	2.21	2.2	0.45	4.74	4.7	0.85	9.46	9.56	1

The second validation involved assessing natural convection in a porous medium, and the current findings were compared with those of Nithiarasu et al.^36^ and Guo et al.^37^ in Table 4. The results demonstrated a favorable alignment with the prior researches, with a maximum error of approximately 4%. This suggests that the current method is well-suited for flows in porous media.

**Table 4:** The average Nusselt number for different Rayleigh number, Darcy number and Porosity in comparison to the prior studies^30,31^.

		Ɛ = 0.4			Ɛ = 0.6			Ɛ = 0.9		
		Present work	Nithiarasu et al.^30^	Guo et al.^31^	Present work	Nithiarasu et al.^30^	Guo et al.^31^	Present work	Nithiarasu et al.^30^	Guo et al.^31^
Da = 10^−2^	Ra = 10^3^	1.016	1.01	1.008	1.017	1.015	1.012	1.024	1.023	–
	Ra = 10^4^	1.368	1.408	1.367	1.499	1.53	1.499	1.638	1.64	–
	Ra = 10^5^	2.978	2.983	2.998	3.416	3.555	3.422	3.864	3.91	–
Da = 10^−4^	Ra = 10^5^	1.069	1.067	1.066	1.073	1.071	1.068	1.075	1.072	–
	Ra = 10^6^	2.578	2.55	2.603	2.718	2.725	2.703	2.747	2.74	–
	Ra = 10^7^	7.815	7.81	7.788	8.245	8.183	8.419	9.151	9.202	–

**Figure 4:**
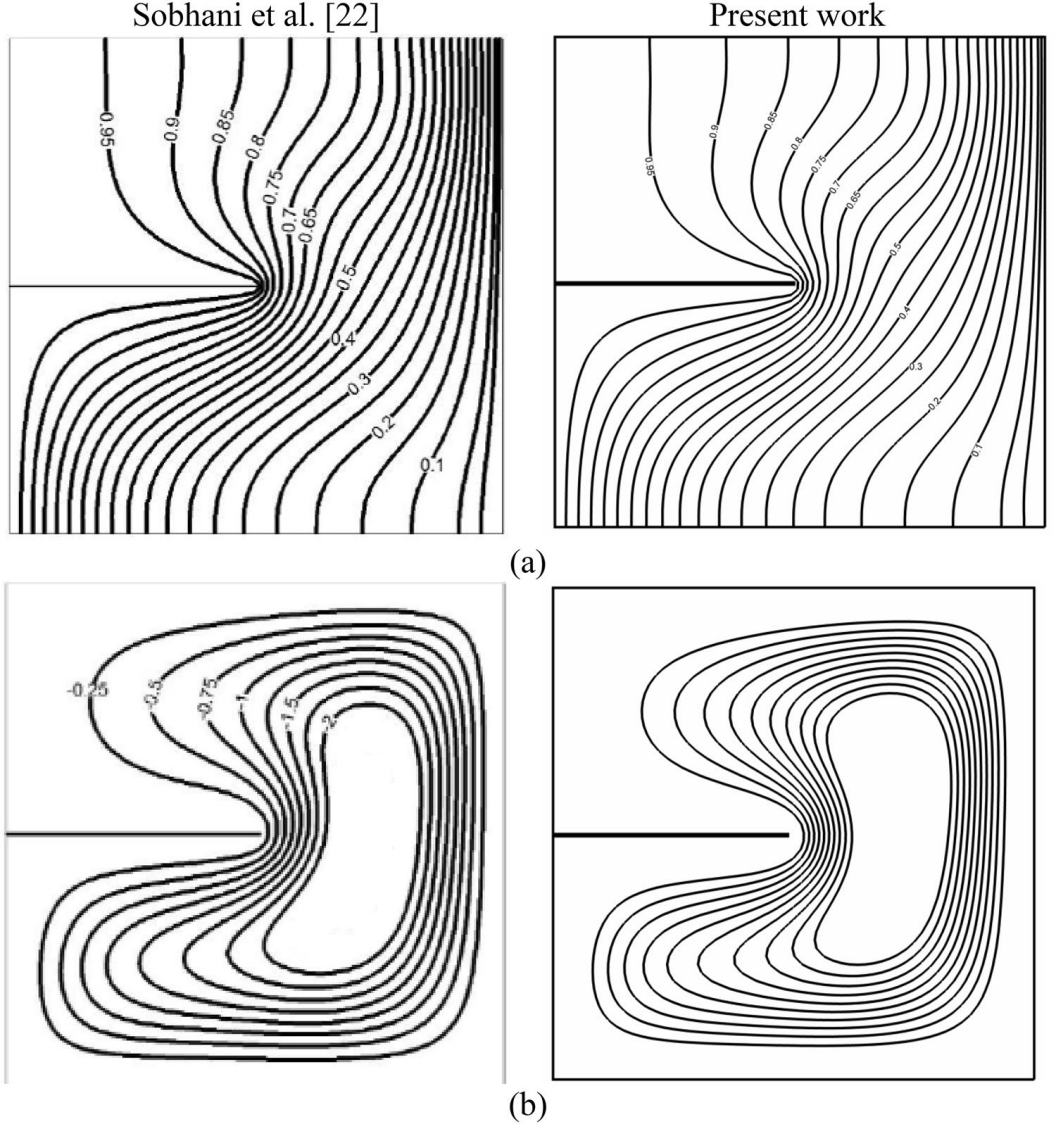
Temperature contours (**a**) and streamlines (**b**) for Ra = 10^4^ in comparison with the previous work^22^.

Also, a qualitative verification of the present work and the previous one^22^ is provided in Fig. 4. As, it is obvious the temperature and velocity field have been captured well.

## Results and discussion

The results of the numerical simulation, the Taguchi method, the Response surface method, optimization, and sensitivity analysis are presented in the following sub-sections. Firstly, obtained design points and the Computational Fluid Dynamics (CFD) results corresponding to these points were presented. Then, TM and RSM were employed to achieve output value in a continuous design space. After that, the optimal points obtained with TM and GA are reported. Finally, the Analysis of Variance (ANOVA) and Global Sensitivity Analysis (GSA) were used to statistically analyze the effect of input parameters on output parameters.

### Design points generation and CFD results

The TM is considered for generating initial design points in the design space. According to the Taguchi design concept, L_27_ standard orthogonal array is used and 27 samples were chosen based on three levels and six factors which are, Length of fins ($H$), number of fins ($N$), distance between fins ($D$), porosity (ε), Darcy number (${\text{Da}}$) and volumetric fraction of the nanoparticles ($\varnothing$). The output parameter at each design point are obtained from MRT-LBM simulations. The input parameters in design points and the corresponding obtained output are presented in Table 5. It will be noted that the final design space includes more than 27 points because 4 new points will be added to improve the final result accuracy.

**Table 5:** Input parameters values of initial design points and the output value obtained from MRT-LBM simulations.

DP	Input parameters						Output parameter
	$H$	$N$	$L$	$\varepsilon$	${\text{Da}}$	$\varnothing$	${{\text{Nu}}}_{avg}$
1	L/6	2	L/6	0.4	0.001	0.005	2.120
2	L/6	2	L/6	0.4	0.010	0.010	3.140
3	L/6	2	L/6	0.4	0.100	0.020	3.410
4	L/6	3	L/8	0.6	0.001	0.005	2.230
5	L/6	3	L/8	0.6	0.010	0.010	3.510
6	L/6	3	L/8	0.6	0.100	0.020	3.852
7	L/6	4	L/12	0.8	0.001	0.005	2.307
8	L/6	4	L/12	0.8	0.010	0.010	3.840
9	L/6	4	L/12	0.8	0.100	0.020	4.258
10	L/3	2	L/12	0.6	0.010	0.005	3.770
11	L/3	2	L/12	0.6	0.100	0.010	4.108
12	L/3	2	L/12	0.6	0.001	0.020	2.607
13	L/3	3	L/6	0.8	0.010	0.005	3.916
14	L/3	3	L/6	0.8	0.100	0.010	4.236
15	L/3	3	L/6	0.8	0.001	0.020	2.703
16	L/3	4	L/8	0.4	0.010	0.005	3.201
17	L/3	4	L/8	0.4	0.100	0.010	3.406
18	L/3	4	L/8	0.4	0.001	0.020	2.434
19	L/2	2	L/8	0.8	0.100	0.005	4.716
20	L/2	2	L/8	0.8	0.001	0.010	2.960
21	L/2	2	L/8	0.8	0.010	0.020	4.535
22	L/2	3	L/12	0.4	0.100	0.005	3.740
23	L/2	3	L/12	0.4	0.001	0.010	2.657
24	L/2	3	L/12	0.4	0.010	0.020	3.694
25	L/2	4	L/6	0.6	0.100	0.005	3.957
26	L/2	4	L/6	0.6	0.001	0.010	2.668
27	L/2	4	L/6	0.6	0.010	0.020	3.884

**Figure 5:**
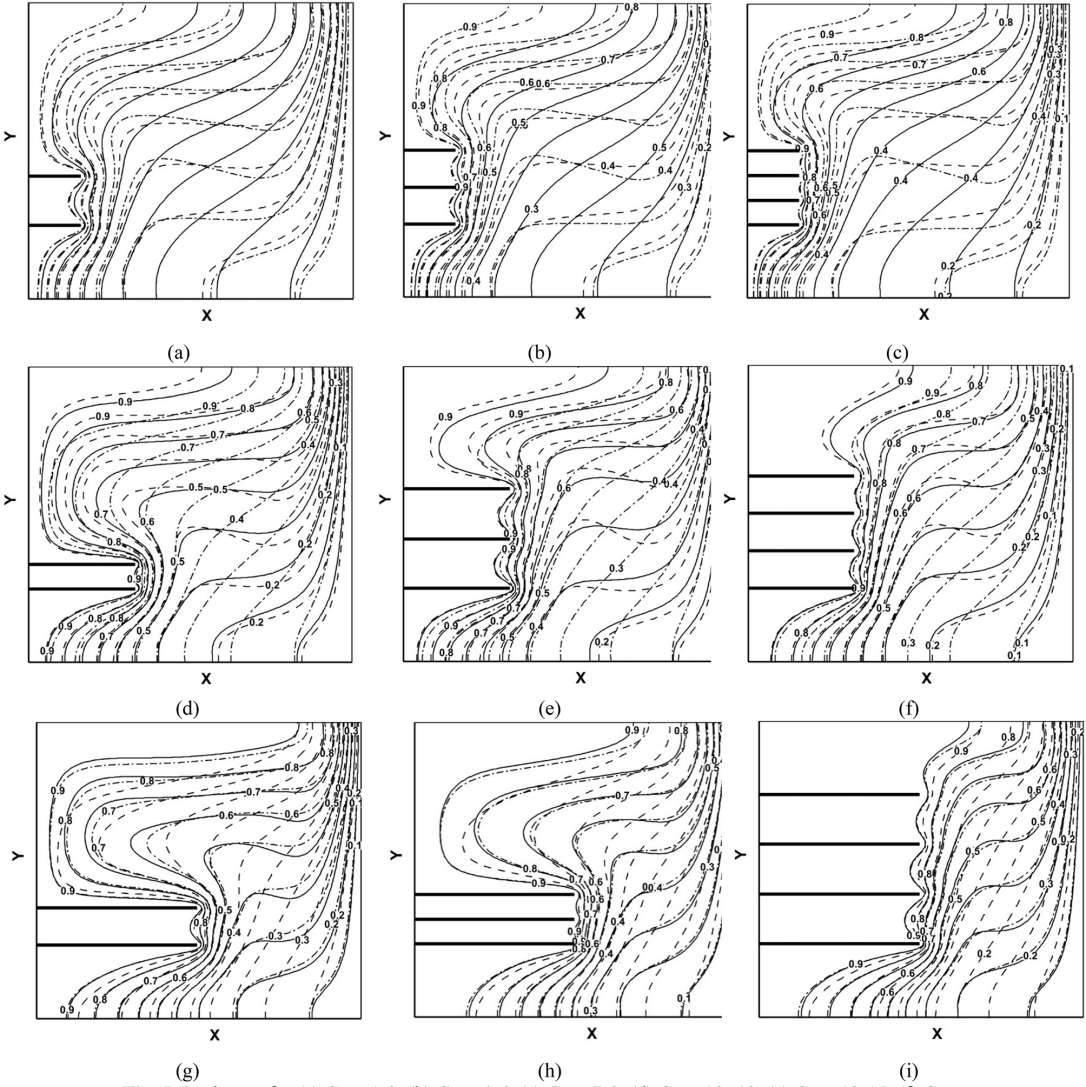
Isotherms for (**a**) Case 1–3, (**b**) Case 4–6, (**c**) Case 7–9, (**d**) Case 10–12, (**e**) Case 13–15, (**f**) Case 16–18, (**g**) Case 19–21, (**h**) Case 22–24, (**i**) Case 25–27-VF of nanoparticles $\varnothing$ = 0.005 solid, $\varnothing$ = 0.01 dashed and $\varnothing$ = 0.02 dashed-dot.

**Figure 6:**
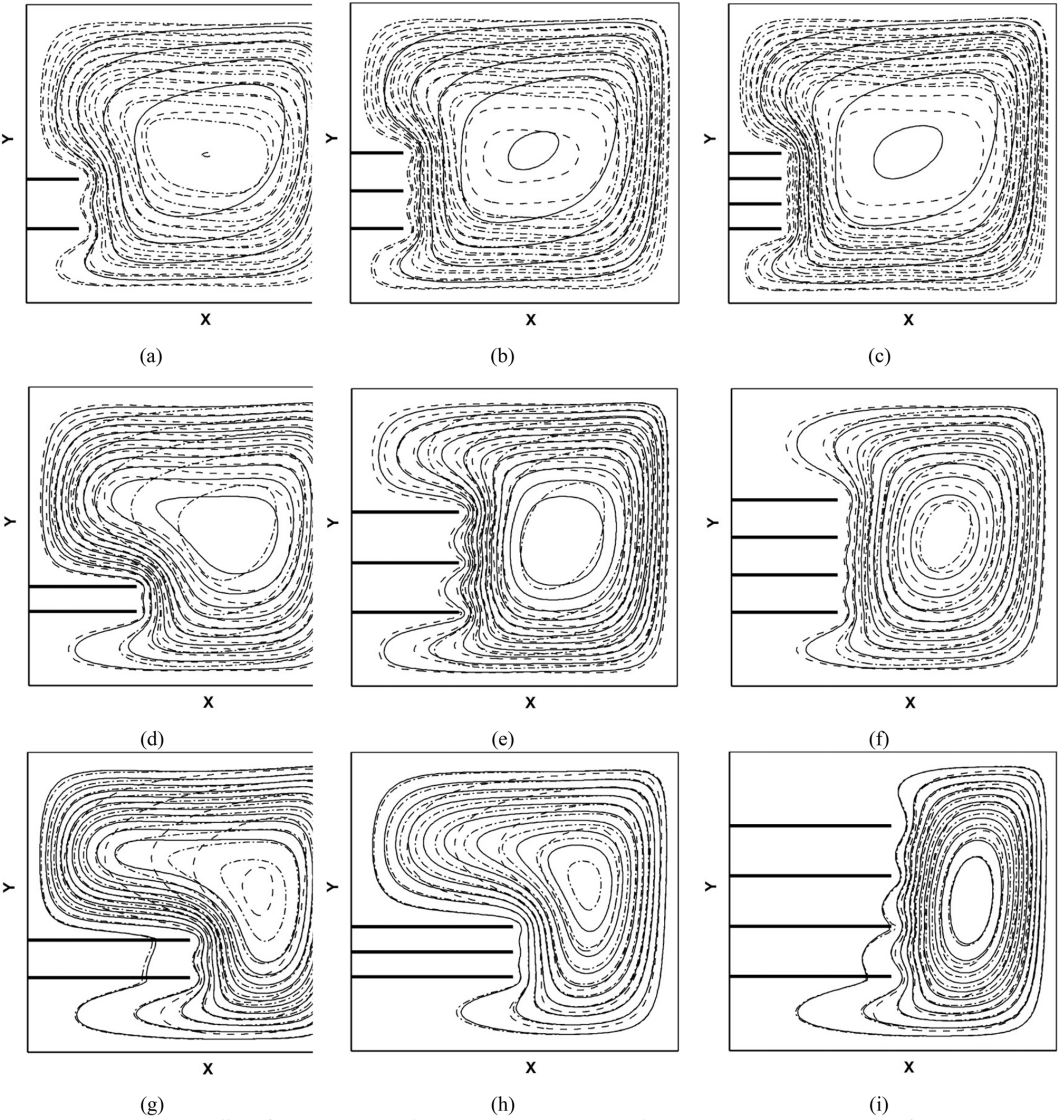
Streamlines for (**a**) Case 1–3, (**b**) Case 4–6, (**c**) Case 7–9, (**d**) Case 10–12, (**e**) Case 13–15, (**f**) Case 16–18, (**g**) Case 19–21, (**h**) Case 22–24, (**i**) Case 25–27-VF of nanoparticles $\varnothing$ = 0.005 solid, $\varnothing$ = 0.01 dashed and $\varnothing$ = 0.02 dashed-dot.

In Figs. 5 and 6, all 27 design points are shown. As can be observed, by changing each of the input parameters, the temperature contours and streamlines change, indicating the effect of these parameters on the heat transfer rate. For example, In Fig. 5 it is noticed by increasing the Darcy number and volumetric fraction of the nanoparticles, the isotherms lines become closer to both hot and cold walls and they become more horizontal which enhances convection in all domains in the cavity. As it was mentioned the Darcy number has a direct relationship with the permeability of the porous medium. By increasing permeability, the resistance to fluid flow is decreased, and velocity, flow recirculation, and thermal convection are enhanced. For low Darcy number heat transfer is primarily governed by conduction within the porous medium, with limited convective heat transfer.

Furthermore, the streamlines (Fig. 6) confirm the above results with expansion of streamlines and rising maximum stream function. Comparing Fig. 5a–c shows that increasing the number of fins and porosity and decreasing distance between them in the stable height of fins, Darcy number and volumetric fraction, increase gradient of temperature near fins bottom-left cornel and upper-right cornel and consequently improve heat transfer. Similarly, comparing Fig. 5a, d and g under equal number of fins, shows when the number of fins is equal to 2, increasing the height of fins helps heat transfer and circulation of flow around the hot wall. However, when the number of fins and the distance between them increases simultaneously, the fluid flow cannot approach the hot wall and as a result heat transfer declines. Moreover, in cases in which the amount of VF of nanoparticles increases, this change improves the conductivity of the working fluid, resulting in higher fluid velocity and more heat transfer from the heated walls to the cooled wall.

In cases with the larger average Nusselt number, the density of streamlines rises. Also in these cases, the streamlines are closer to the hot wall and the circles of flow can be in touch with the hot wall and back to sink.

### TM and RSM results

**Figure 7:**
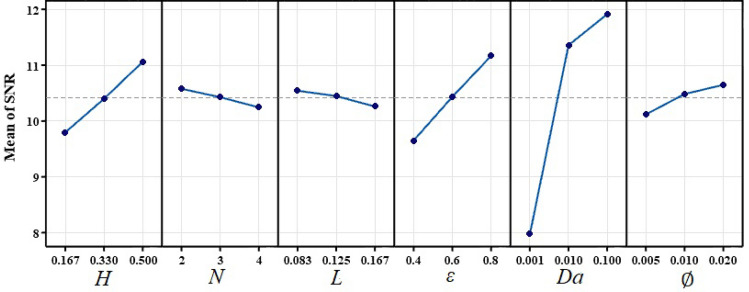
SNR for each factor at each level.

From the numerical simulation results and using the TM at first the discrete design space will be converted to a continuous design line in orthogonal arrays. An advantage of SNR is obtaining the levels of parameters that enhance heat transfer. Figure 7 shows the results of SNR values for each factor at each level. Based on the TM, the level with the highest SNR value corresponds to the optimal value of the control parameter. Results show that the increasing length of fins ($$H$$), porosity (ε), Darcy number ($${\text{Da}}$$) and volume fraction of nanoparticles ($$\varnothing $$) increases the output parameter. However, when the number of fins and distance between ($$D)$$ them rise, the outlet decreases.

By using RSM, the discrete design space will be converted to a continuous design space. RSM creates the approximation functions by some mathematical and statistical tools to describe the design space. Here, design points are those obtained in the previous step (Taguchi method DOE) and the samples which are added to make better predictions (Table 6). Also, the GA method has been taken for regression and predicting output parameter in the whole design space.

**Table 6:** Added design points to improve response surface method results.

DP	Input parameters						Output parameters
	$H$	$N$	$L$	$\varepsilon$	${\text{Da}}$	$\varnothing$	${{\text{Nu}}}_{avg}$
1	5L/12	2	L/9	0.7	0.05	0.015	4.446
2	5L/12	3	L/9	0.7	0.05	0.015	4.386
3	5L/12	4	L/9	0.7	0.05	0.015	4.295
4	L/2	2	L/12	0.8	0.1	0.02	5.56

To obtain a high-quality response surface, another level is created in the design space. With this method, conditions are created so that the response surface equation can have a higher order to create better predictability and curve fitting. In the previous step, there were only three levels that were suitable for fitting the curve with the quadratic polynomial. Table 6 shows the sample design points which added to the initial design points (Table 5).

**Figure 8:**
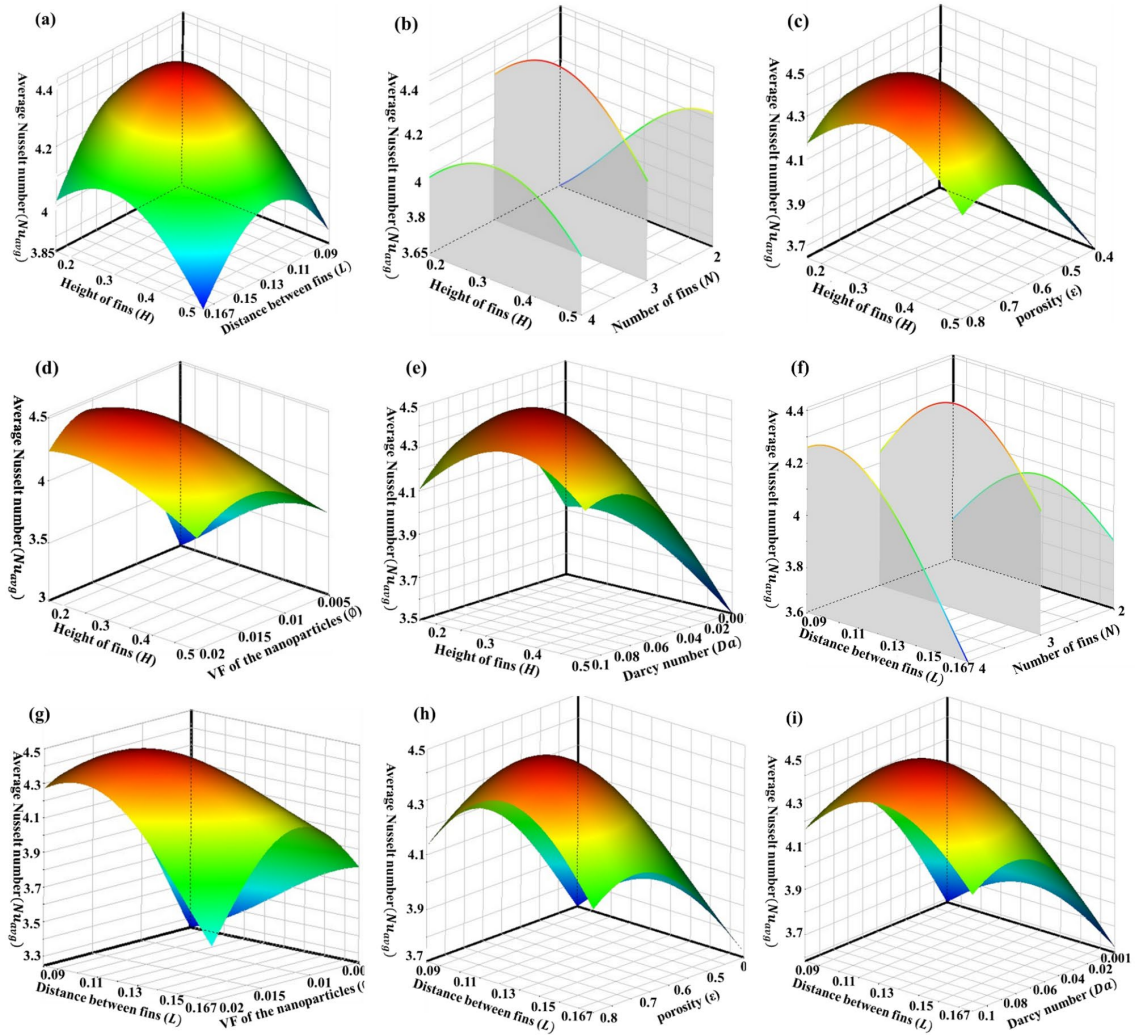
3D Response surfaces for the average Nusselt number as a function of input parameters.

Figure 8 shows the 3D response surface for the average Nusselt number on the cold wall as the function of input parameters. Note that in each plot, the output parameter is shown with two input parameters while the other input parameters have been considered constant at the mean value of its design range.

Figure 8a and b show that configuration and number of fins have a significant effect on the output parameter. For example, in Fig. 8a it is noticeable that the average Nusselt number can change from 3.85 when the length of fins is 0.5 and the distance between fins is 0.167 to more than 4.4 when length of fins and distance between them are in the middle of their change interval. Also, when the length of fins increases the behavior of the output parameter is very different in each number of fins. Figure 8c shows that when the porosity of the porous cavity increases approximately in each length of fins, the average Nusselt number goes up. This trend is for increasing of volume fraction of nanoparticles and Darcy number, too.

When the changeable parameters are the distance between fins and the number of them, as it is obvious in Fig. 8f, the output parameter is dependent on the input parameters significantly. For instance, when the number of fins is equal to 4, increasing the distance between fins causes a sharp decrease in the Nusselt number. This is probably because by increasing the distance between the fins, the flow cannot approach the hot wall and vortices are not allowed to form. However, when the number of fins is equal to 2 and 3 the average Nusselt number first increases and then decreases with the increase of the distance between the fins. It is clear that by rising porosity, the volume fraction of nanoparticles and Darcy number, generally average Nusselt number increases. Interestingly, the response of output parameter to porosity and Darcy number is approximately similar.

**Figure 9:**
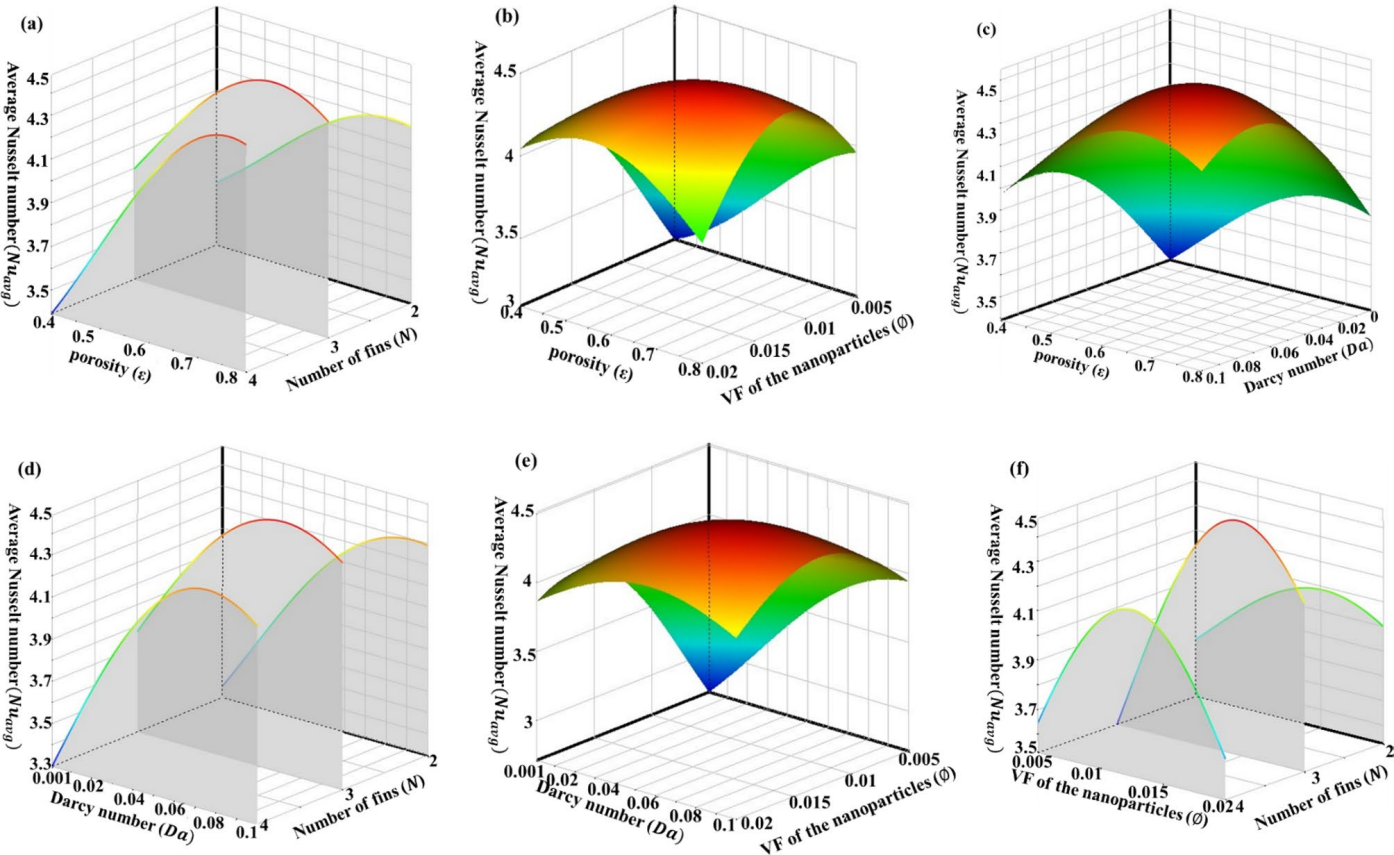
3D Response surfaces for the average Nusselt number as a function of input parameters.

Figure 9a shows that by increasing porosity, in each number of fins, ${{\text{Nu}}}_{avg}$ will be increased when other input parameters are in the middle of their change interval. The results show that when the porosity and volume fraction of nanoparticles are changeable parameters in the problem, the maximum value of the ${{\text{Nu}}}_{avg}$ is placed somewhere in the middle of the range of changes (tending to the upper limit) (Fig. 9b). But when the porosity is high and the volume fraction of nanoparticle is low or vice versa, this value decreases slightly. In Fig. 9c, it can be seen that the increase of Darcy and Prosity number increases the output function. Also, at any fixed number of fins, the output increases with the increase of the Darcy number (Fig. 9d). It is noticeable in Fig. 9e. When Darcy number and volume fraction of nanoparticles are variables, the increase of both parameters increases the output, however, the effect of the first parameter is greater. It is obvious in Fig. 9f that by increasing the volume fraction of nanoparticles to about 0.015, in each number of fins, first ${{\text{Nu}}}_{avg}$ will be increased then for more volume fraction of nanoparticles the heat transfer rate will decrease.

The results show that, generally, increasing porosity enhances heat transfer, except for very high porosity, in which heat transfer slightly decreases. This may occur because the conductivity decreases in high porosity. However, low porosity is associated with a high pressure drop and requires significant pumping power.

### Optimization

In this section, the optimal points obtained by the TM and GA (based on the results of design points and predictions of RSM) where the ${{\text{Nu}}}_{avg}$ is maximum are reported. In addition, the points where this output parameter is minimum are also given. Table 7 indicates the optimum point in which the average Nusselt number is maximized and also the point where this parameter minimized.

**Table 7:** Optimum point from TM.

Parameters	Length of fins ($H$)	Number of fins ($N$)	Distance between fins ($L$)	Porosity ($\varepsilon$)	Darcy number (${\text{Da}})$	VF of the nanoparticles ($\varnothing )$	${{\text{Nu}}}_{avg}$ (predicted by Taguchi)	${{\text{Nu}}}_{avg}$ (calculated by LBM)	TM- LBM difference (%)
Maximum point (optimum condition)	L/2	2	L/12	0.8	0.1	0.02	4.88	5.56	12.23
Minimum point	L/6	4	L/6	0.4	0.001	0.005	1.996	2.08	4.038

Utilizing the RSM approach, the established continuous design space enables optimization with notable reductions in computational expenses. The GA is applied to optimize the output parameter, and the corresponding outcomes are presented in Table 8. The conditions selected by the GA are very close to the point selected by the TM. The optimum point predicted by the TM showed a higher average Nusselt number. However, the error rate in Taguchi’s prediction was almost more compared to the prediction of the GA.

**Table 8:** Optimum point from GA.

Parameters	Length of fins ($H$)	Number of fins ($N$)	Distance between fins ($L$)	Porosity ($\varepsilon$)	Dar cy number (${\text{Da}})$	volumetric fraction of the nanoparticles ($\varnothing )$	${{\text{Nu}}}_{avg}$ (predicted by GA)	${{\text{Nu}}}_{avg}$ (calculated by LBM)	GA-LBM difference (%)
Maximum point (optimum condition)	L/2	2	L/9	0.8	0.1	0.02	5.26	4.899	7.3
Minimum point	L/6	3	L/9	0.5	0.001	0.005	2.10	2.19	4.1

**Figure 10:**
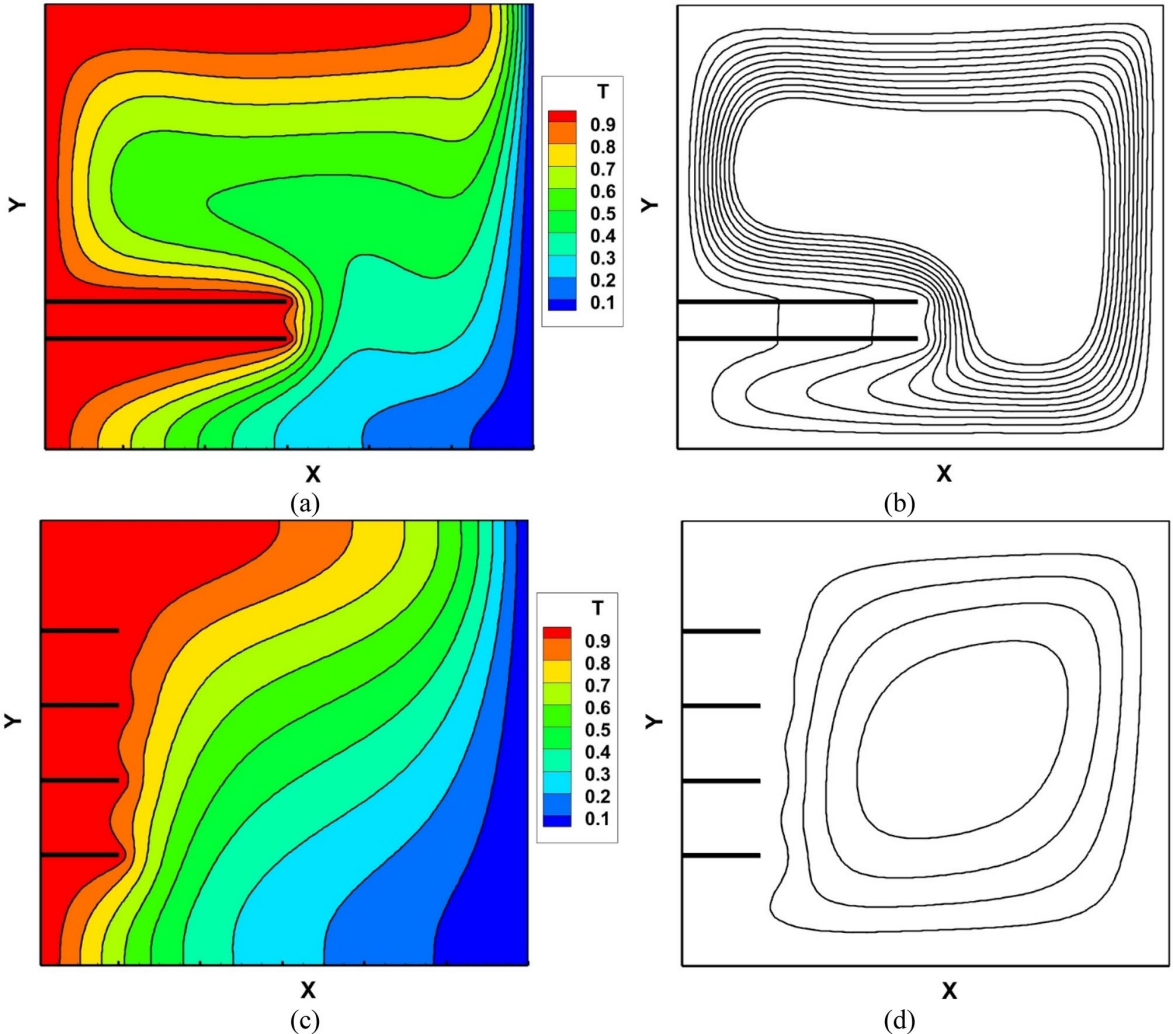
Temperature contour (**a**) maximum point (**c**) minimum point and streamlines (**b**) maximum point (**d**) minimum point obtained based on TM.

**Figure 11:**
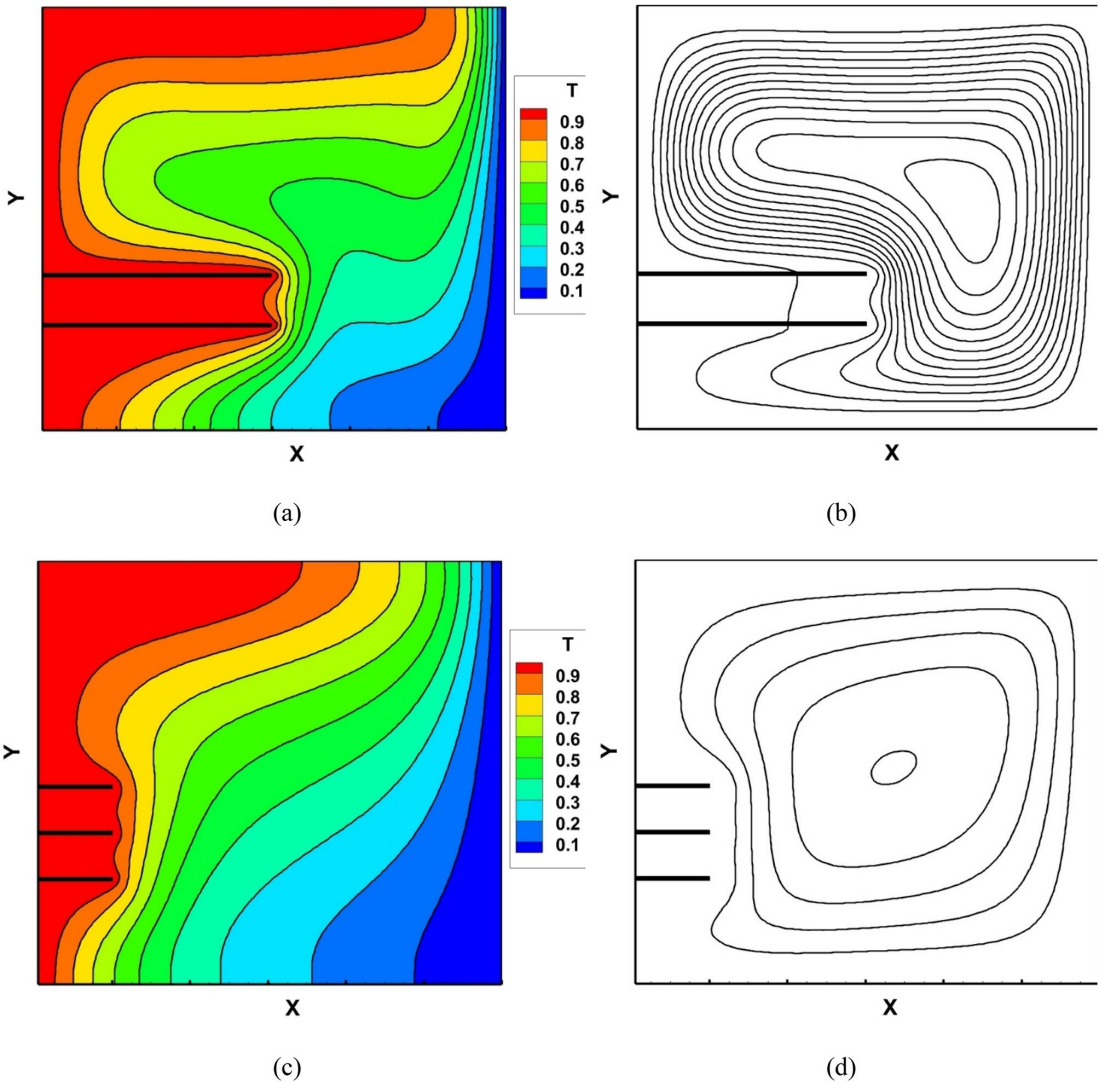
Temperature contour (**a**) maximum point (**c**) minimum point and streamlines (**b**) maximum point (**d**) minimum point obtained based on GA.

Figure 10 shows isotherm and streamlines for the optimum point obtained by the TM. For the optimum condition, as determined from the temperature contour (Fig. 11a), the temperature gradian next to the hot and cold wall is much higher compared to the worst condition (Fig. 10c). The density of streamlines in Fig. 11b, which shows optimum point, is really high compared to when ${{\text{Nu}}}_{avg}$ is at its minimum value (Fig. 11d).

Figure 11 shows isotherm (Fig. 11a,c) and streamlines (Fig. 11b,d) for optimum point obtained by GA. The things that were mentioned for the minimum and maximum points obtained by the TM can also be seen here. Since the optimal point obtained by this method (Fig. 11a,b) is geometrically very similar to the previous optimal point (Fig. 10a,b), the temperature contour and their streamlines are almost similar.

### Sensitivity analysis

In this section, ANOVA and GSA are used to statistically investigate the effect of independent parameters on the output parameter. The type of ANOVA is used in this part is ANOVA by DOE which these design points are based on the same points that were used in the TM (L_27_ orthogonal arrays). But GSA was applied to the available data in RSM.

As mentioned in section "Taguchi method (TM) and response surface method (RSM)", ANOVA can be used to illustrate how the variables affect the output parameter by using the ratio of the variance of each variable to total variance. The larger *F* value (the ratio of two variances) shows that variation of input factor plays major role in performance of response parameter. The percentage of contribution is calculated by dividing *F* value of each section in total *F* value. Table 9 shows Darcy number by far is the most effective parameter with a contribution ratio of 79.37%. Following by porosity and length of fins in second and third place with a contribution ratio of 9.83% and 6.63%, respectively. Other parameters have negligible effect on the output parameter with a contribution of less than 2%.

**Table 9:** ANOVA results for the Nusselt number.

Parameters	Degree of freedom	Seq SS	Adj MS	F	Contribution (%)
Length of fins ($H$)	2	7.047	3.523	33.566	6.63
Number of fins ($N$)	2	0.516	0.258	2.459	0.30
Distance between fins ($L$)	2	0.363	0.182	1.731	0.15
Porosity ($\varepsilon$)	2	10.336	5.168	49.235	9.83
Darcy number (${\text{Da}})$	2	81.997	40.998	390.593	79.37
Volumetric fraction of the nanoparticles ($\varnothing )$	2	1.315	0.657	6.263	1.07
Residual error	14	1.470	0.1041		
Total	26	103.043			

**Figure 12:**
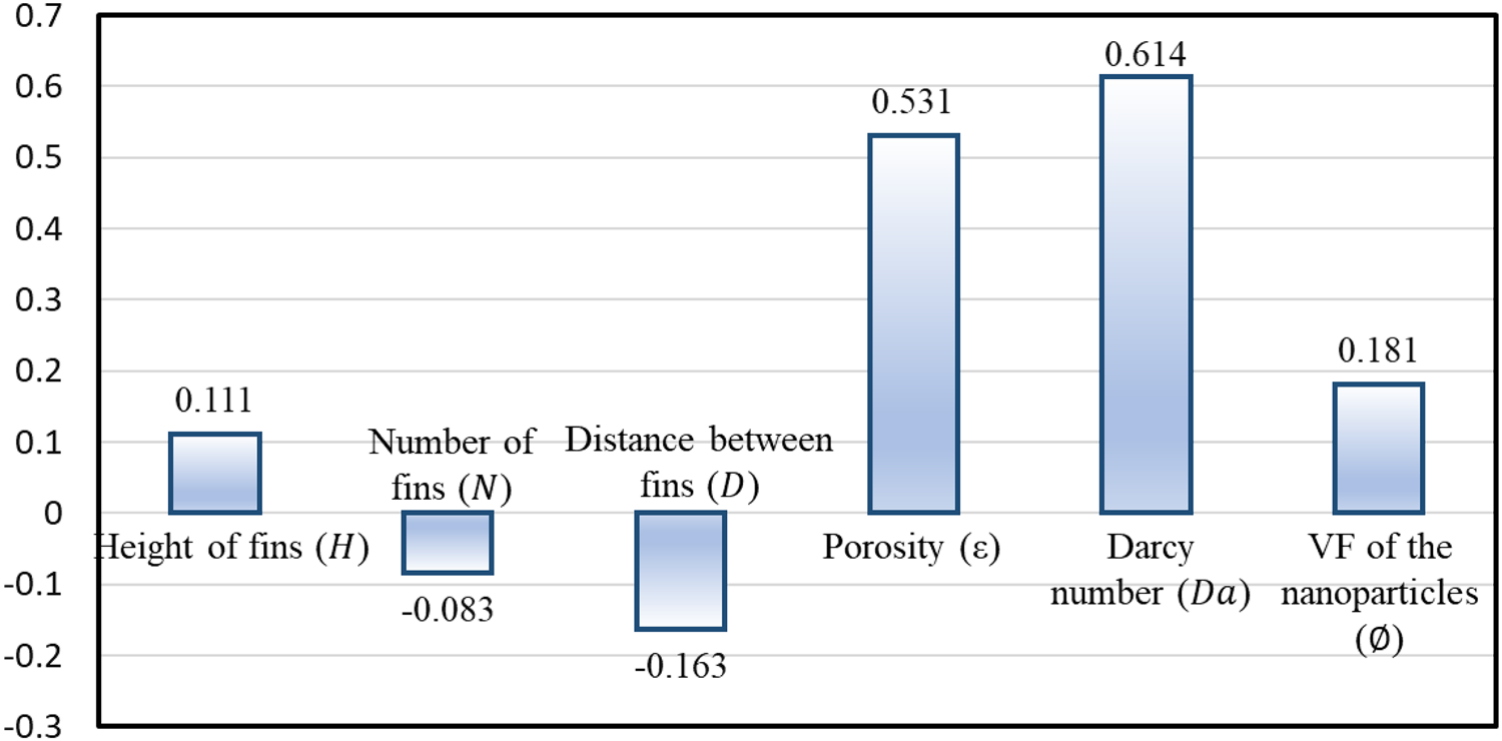
Global sensitivity of the input–output parameters.

Due to the complicated interaction between input parameters and the output response, employing a GSA is valuable for assessing the importance of the design parameters. GSA involves simultaneously varying all input factors and assessing sensitivity across the entire range of each input variables. Figure 12 illustrates the outcomes of the analysis. It should be noted that the positive values of the sensitivity analysis indicate that the response increases as the value of the input parameter increases. The negative sensitivity factor displays an inverse correlation in this relation. According to the results, it is obvious that Darcy number is the most effective parameter with the factor of 0.614. The second influential parameter on the output is porosity.

## Conclusion

The Taguchi method (TM) and genetic algorithm (GA) contributed greatly to reduce cost of numerical simulation for better understanding fluid flow and heat transfer in porous cavity filled by nanofluids. In this paper, by creating a design space consisting of six input parameters, included length, number and distance between fins, porosity, Darcy number and volumetric fraction of nanoparticles, an attempt was made to reach the appropriate physical and geometrical conditions to obtain maximum average Nusselt number. First, to reducing number of design points of experiments (DOE), TM was utilized. Then the double MRT-LBM was carried out on 27 initial design points generated by TM. After that, using the MRT-LBM results, TM and GA, the output parameter were answered in the entire design space. Regarding the obtained response surfaces, an optimization was accomplished and the sensitivity of the output variable from the input variables was investigated. According to the results, the following conclusions were drawn:

- The best desing for increasing the heat transfer rate is: The length of fins ($H$= L/2), the number of fins ($N=$ 2), the distance between fins ($D$= L/12), porosity ($\varepsilon =$ 0.8), Darcy number (${\text{Da}}=$ 0.1), and volumetric fraction of the nanoparticles ($\varnothing =$ 0.02).
- The worst position for heat transfer enhancement is: The length of fins ($H=$ L/6), the number of fins ($N=$ 4), the distance between fins ($D=$ L/6), porosity ($\varepsilon =$ 0.4), Darcy number (${\text{Da}}=$ 0.001), and volumetric fraction of the nanoparticles ($\varnothing =$ 0.005).
- Darcy number is the most effective parameter compared other variables studied in his work on average Nusselt number with GSA 0.614 and ANOVA contribution of 79.37%.
- Number of fins is the least effective parameter compared other variables studied in this work on heat transfer rate with GSA -0.083 and ANOVA contribution of 0.3%.
- Prediction of GA is more accurate than the TM in comparison with the simulation results.

## Data Availability

The datasets used and/or analyzed during the current study available from the corresponding author on reasonable request.

## List of symbols

${f}_{i}$: Velocity distribution function
${h}_{i}$: Temperature distribution function
$$K$$: Permeability
$$V$$: Volume
$$g$$: Gravity
$$k$$: Thermal conductivity
P: Pressure
T: Dimensionless temperature
$${T}{\prime}$$: Temperature
$${\text{Da}}$$: Darcy number
$${\text{Nu}}$$: Nusselt number
Ra: Rayleigh number
X, Y: Dimensionless coordinates
M: Transformation matrix for flow field
N: Transformation matrix for temperature field
U-V: Dimensionless x–y directions coordinate velocity
u–v: X–y directions coordinate velocity
SNR: Signal-to-noise ratio

## Greek symbols

$$\alpha $$: Thermal diffusivity
$\beta$: Thermal expansion coefficient
$$\rho $$: Density
$$\varepsilon $$: Porosity
$$v$$: Kinematic viscosity
$$\mu $$: Dynamic viscosity

## Subscripts

$$c$$: Cold
$$h$$: Hot
$$avg$$: Average
$i,j$: Indexes for the discrete direction
$x, y$: X- and y-coordinate system
$$s$$: Solid
$$nf$$: Nanofluid
$$f$$: Fluid
